# Rac1 GTPase in pancreatic cancer

**DOI:** 10.18632/aging.100804

**Published:** 2015-09-27

**Authors:** Ying Yan, Michel M. Ouellette

**Affiliations:** Department of Internal Medicine, Division of Gastroenterology and Hepatology, University of Nebraska Medical Center, Omaha, NE 68198, USA

**Keywords:** Rac1, pancreatic cancer, K-Ras, GTPase, chemotherapy, radiotherapy

**Figure 1:**
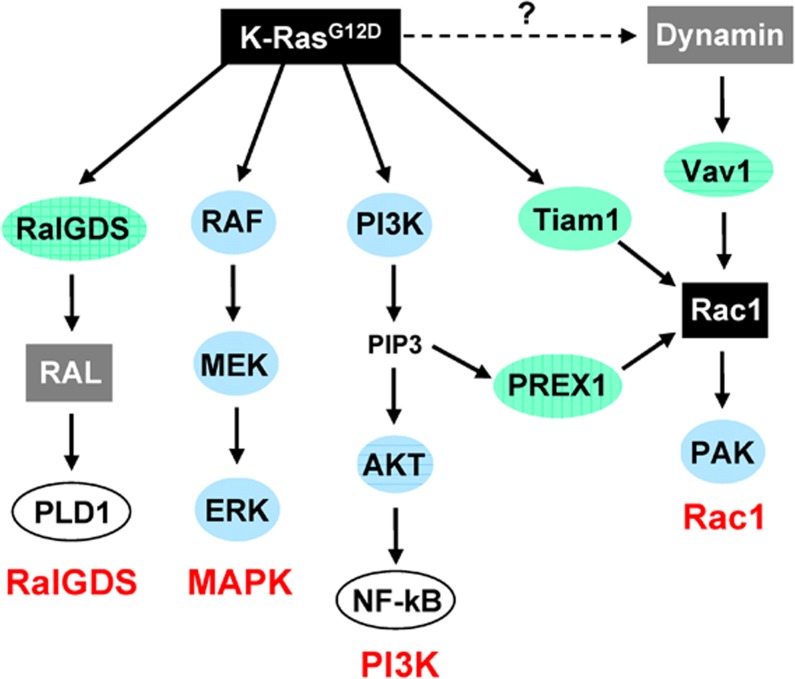
Effectors of oncogenic K-Ras K-Ras signals through activation of its downstream effectors: the Ral-GDS, MAPK, PI3K, and Rac1 pathways. Several independent pathways are responsible for Rac1 activation in pancreatic cancer cells, many of which under the control of oncogenic K-Ras (Tiam1, PREX1).

Pancreatic cancer is the fourth leading cause of cancer death in the Western world. It is a disease of insidious progression and high lethality, with a 5 year survival rate of only 6%. In spite of significant advances in pancreatic cancer research, current therapies are ineffective and fail to significantly extend lifespan. Thus, there is an urgent need for novel and improved therapies for this disease. Activating mutations in the K-Ras gene are the earliest and most common genetic alterations detected in pancreatic cancer specimens. Efforts to target K-Ras with small molecule inhibitors have not yet met with success, and an alternative strategy has been to target its downstream effectors. K-Ras has four effectors that play a role in cancer development (Fig. 1): mitogen-activated protein kinase (MAPK) pathway, phosphoinositide 3-kinase (PI3K) pathway, Ral guanine nucleotide dissociation stimulator (Ral-GDS), and Ras-related C3 botulinum toxin substrate 1 (Rac1) GTPase. Efforts to block these effectors have so far been focused on the MAPK and PI3K pathways, mainly because of the availability of drugs to target these pathways. Studies have now highlighted the importance of yet another K-Ras effector, the Rac1 signaling pathway. This pathway, which has not yet been extensively studied, is as critical for PC development as the MAPK and PI3K pathways.

Rac1 is an isoprenylated membrane-bound protein that belongs to the Rho family of small GTPases [1]. Like K-Ras and other GTPases, Rac1 can exist in either an inactive GDP-bound state or active GTP-bound form. In its active form, Rac1 interacts with its downstream effectors, activating them in the process. Many of Rac1 effectors are proteins involved in remodeling the actin cytoskeleton and regulation of cell motility and migration, such as the WAVE and Arp2/3 complexes. Other effectors, like the PAK kinases, have been implicated in the regulation of MAPK signaling, survival and proliferation. The level of GTP-bound Rac1 is controlled by the activities of guanine nucleotide exchange factors (GEF), GTPase activating proteins, and guanine dissociation inhibitors. Two of the GEFs responsible for Rac1 activation, Tiam1 and PREX1, are themselves stimulated by the Ras oncogenes (Fig. 1). Rac1 is also activated by the GEF Vav1 (Fig.1), and Vav1 is up-regulated in the majority of pancreatic cancers. While levels of GTP-bound Rac1 have never been measured in pancreatic cancer specimens, the pathway is generally assumed to be activated in these tumors, at least in part by the presence of activated K-Ras.

Recent studies have highlighted the role of Rac1 in the initiation and progression of pancreatic cancer. In mouse models of pancreatic cancer, the pancreas specific activation of K-Ras leads to acinar-to-ductal metaplasia (ADM) and formation of PanIN precursor lesions. In this model, the pancreas-specific ablation of the Rac1 gene abrogates the development of ADM, delays the formation of PanIN lesions, and blocks progression to pancreatic cancer [2]. These observations demonstrate the importance of Rac1 signaling in pancreatic cancer development, but they have not yet addressed its role in tumor maintenance or potential as a therapeutic target. Recent studies have used interfering RNA, pharmacological inhibitors, or dominant negative mutant of Rac1 (Rac1^T17N^) to block Rac1 signaling in cultivated pancreatic cancer cells. Consistently, these interventions have led to reductions in cell proliferation, viability, and migration. In mice implanted with pancreatic tumors, intratumoral injections of an adenovirus expressing the Rac1^T17N^ mutant have also led to significant tumor growth inhibitions. Recent studies have also revealed an unexpected role for Rac1 in the response of cancer cells to DNA damaging agents. In breast and pancreatic cancer cells, Rac1 inhibition blocks activation of a G2/M cell cycle checkpoint that protects cells from the effects of IR and radiomimetic agents [3, 4]. These and other findings have revealed the potential value of Rac1 pathway inhibition in cancer therapy.

The next step in the development of Rac1 pathway inhibitors as chemotherapeutic agents will be the testing of second generation Rac1/PAK inhibitors and development of next generation inhibitors. The first small molecule inhibitors of the pathway were the Rac1 inhibitors NSC23766 and EHT-1864, and the PAK kinase inhibitor IPA-3. These compounds have constituted invaluable tools in studies of the role of pathway in cancer cells, but were not sufficiently potent for animal studies. Second generation inhibitors that block the pathway at the lower micromolar range (1-5 μM) have now been described. These new compounds include the Rac1 inhibitors Ehop-016 (blocks Rac1 and Rac3) and AZA1 (blocks Rac1 and Cdc42) and PAK kinase inhibitor FRAX597 (blocks group I, but not group II, PAK kinases). These drugs have been reported to produce significant tumor growth inhibition in mouse models of breast, prostate, and brain cancer, respectively [5-7]. None of these inhibitors have yet been tested in preclinical models of pancreatic cancer. Futures studies should soon determine if these drugs have potential value in the treatment of pancreatic cancer and other Ras-driven malignancies.
